# Averting Potential Misinterpretations and Diagnostic Misapplications of Misleading Laboratory Results

**DOI:** 10.3390/diagnostics16162527

**Published:** 2026-08-11

**Authors:** Adel A. A. Ismail

**Affiliations:** Clinical Biochemistry and Chemical Endocrinology, Mid-Yorkshire and Leeds Teaching Hospitals, Wakefield WF2 6HL, UK; adelaaismail@aol.com

**Keywords:** diagnostic misapplication, chaos, Bayesian, immunoassay’s misinterpretation, analytical errors

## Abstract

Diagnosis is a dynamic process, encompassing clinical presentation of pathophysiology, workup investigations and appropriate interpretations/integration of all data. Diagnostic accuracy is expected when based on multiple tests/investigations. However, when diagnosis is based on a single abnormal laboratory test, considered to be fundamental and deterministic, an early test’s error could cascade and propagate, triggering unnecessary investigations, longer hospitalisation and possible diagnostic misapplications. Up to 70% of diagnoses involve laboratory tests. Immunologically based analyses (immunoassays) have two fundamental features: (a) inherently higher analytical error rate than other common routine tests such as U&E, LFT, bone profile, FBC, et cetera, and (b) universality, affecting measurements regardless of the analyte’s nature or the immunoassay’s provider. Averting potentially erroneous results necessitates a paradigm shift which can be made by (a) intuitive use of a statistical paradigm (Bayesian reasoning), which will be explained by a simple example and without equations, and (b) appropriate and more use of follow-up confirmatory tests when the test has consequences and potential diagnostic misapplications by using available tests in all hospital laboratories.

## 1. Introduction

Appreciation of the long-term consequences of an early error and their impact on an evolving dynamic process has its roots in Chaos mathematics, a new branch discovered by chance in the 1960s by the mathematician/meteorologist Edward Lorenz. Early computers at that time had limited “private” internal code memory that computed calculations. Edward Lorenz rounded “public” printed number readings from six decimal places to just three to simplify calculations, assuming this to be inconsequential (being one part in a thousand). To his surprise, such an assumption of slight differences turned out to have a catastrophic impact on long-term output forecasted by these two different methods [1]. The impact of initial/early error was subsequently described by Edward Lorenz at the annual meeting of the American Association for the Advancement of Science in Washington in 1972 in a metaphor commonly known as the “Butterfly Effect”, i.e., tiny air turbulence caused by a Butterfly flapping its wings in Brazil propagates and sets off a tornado in Texas. Chaos mathematics emerged as dynamic systems that can be sensitive to an initial error. The analogy to clinical diagnosis may be perceived as loose/tenuous because clinicians are intuitively aware of such a concept; this problem, however, continued to occur with early error being overlooked, causing chaos and confusion, wasting time and resources not only clinically but also forensically, too. The aim of this review is to describe (a) the clinical impact of analytical errors on the misinterpretation of laboratory data by focusing on details of a specific example, briefly followed by some other (b) Bayesian approaches, which could help identifying such potential errors and, thus, help averting its adverse effects and (c) explain the underlying causes of high error rate in immunoassays and, finally, (d) describe follow-up tests which could identify false and erroneous analytical results available in all hospital laboratories.

## 2. Search Strategy and Evidence Selection

This review was not conducted as a formal systematic review or meta-analysis of laboratory testing methodologies. Rather, it is a narrative review intended to highlight the relatively higher frequency of analytical errors associated with immunoassays compared with other routinely performed tests in clinical biochemistry and hematology. The analytical error is method- and reagent-dependent; hence, no systematic or multicentre studies were carried out. Because of this, this review incorporates illustrative case reports and clinical examples that demonstrate the potential clinical consequences of unverified abnormal immunoassay results across a range of medical conditions.

A comprehensive literature search was undertaken using PubMed, MEDLINE, and Scopus databases, supplemented by manual screening of reference lists from relevant publications of case reports. The review primarily considered peer-reviewed articles published in English between January 2000 and December 2025. However, selected publications published before 2000 were also included when deemed particularly relevant to the topic. Search terms included combinations of the keywords: *immunoassay errors*, *immunoassay interference*, *misinterpretation of immunoassay results*, *misleading immunoassay results*, *analytical errors*, and *wrong diagnosis*. Articles were selected based on their relevance to the occurrence, mechanisms, clinical implications, and diagnostic consequences of immunoassay-related analytical errors when accepted at face value without verification of the integrity of analyses.

## 3. Clinical Impact of an Early Error

The impact of an early error on diagnosis may be best illustrated (i) by describing the confusion caused by analytical laboratory errors in investigating protracted/persistent hypoglycaemia of unknown origin, in non-diabetic patients and (ii) by other analytical errors of immunoassays affecting other analytes and their consequences.

### 3.1. Hypoglycaemia of Unknown Origin in Non-Diabetic Patients

Current practice involves taking a blood sample during the hypoglycaemic phase for the measurement of insulin and C-peptide. Initial findings of high C-peptide concentrations associated with high insulin levels during hypoglycemia suggest an endogenous source, e.g., insulinoma or hyperinsulinemic hypoglycemia. Furthermore, an undetectable/low C-peptide associated with high insulin levels raises suspicions of exogenous insulin administration, i.e., factitious hypoglycemia. A molar ratio of insulin/C-peptide > 1.0 supports such interpretation [2] (physiological setting, molar ratio is ≤0.2).

Extending the use of C-peptide as an index of endogenous pancreatic secretion from diabetics to non-diabetics with an undetermined cause of hypoglycaemia is simplistic, confusing and could be misleading. For example, the variability of C-peptide levels in pathologies is demonstrated in the unique study of a Dutch case with hypoglycaemia [3] in whom blood samples were taken every 3 h for 48 h (total 17 samples). Expected fluctuations in serum Insulin concentrations in response to meals occurred, but levels remained very high throughout. In 10 of the 17 samples, however, C-peptide concentrations were within the reference range, yet were undetectable in seven other samples, which would have been suggestive of exogenous insulin administration if a single blood sample was taken for analysis during any of these times. Confirmatory tests showed a high titre of insulin autoantibodies (IAAs) which bind insulin, confirming the diagnosis of insulin autoimmune syndrome (IAS), a condition also known as Hirata’s disease.

Hirata’s disease (IAS) is a unique autoimmune disorder in which endogenously produced autoantibodies bind insulin (IAA) and, in rare cases, insulin receptors [4]. This IAA has a double whammy effect, causing hypoglycaemia and high insulin results, with normal or low/undetectable C-peptide. This condition was first discovered in a Japanese adult by Yukimasa Hirata. It was initially published in Japanese in *J Jpn Diabetes Soc* in 1970 [5] and subsequently in English in *Tohoku Journal of Experimental Medicine* in 1972 [6]. The IgG classes of IAA have a long t_1/2_ of approximately 3–4 weeks. Of interest also, the first case of a newborn with hypoglycaemia caused by IAA was discovered and reported around the same time by Nakagawa and published in 1973 [7]. The newborn was a Japanese baby who had protracted hypoglycaemia and high IAA titre (IgG class), transferred during pregnancy from the mother (secondary IAS). Surprisingly, however, the baby’s mother (25 years old) did not suffer from any hypoglycaemic episodes, yet had the same high titer of IAA (IgG class) as well as extremely high serum insulin levels. This highlighted the wide variation in symptoms and insulin measurements caused by endogenous IAAs. It is now recognised that IAS is common in individuals with human leukocyte antigen [2,8] (HLA) types such as DR4, DRB1 ∗ 0406, DRB1 ∗ 0403, DQB1 ∗ 0302, DQA1 ∗ 0301, DRB1 ∗ 0415, and DRB1 ∗ 1301. The three major triggers of IAS in these predisposed individuals [2,8] are drugs, the presence of autoimmune disease(s) and viral infection/reinfection, e.g., mumps, rubella, Coxsackie B influenza, hepatitis C, chickenpox and measles. The innate immune response to infection/reinfection normally triggers autoantibody production by B-cells, which rise exponentially (i.e., increase slowly at first, followed by a rapid rise, doubling every few hours). Drugs, especially those containing a sulphydryl (thiol) group or which produce this structure during metabolism, could also trigger IAS; examples are methimazole, pyritinol, glutathione methionine, 2-mercaptopropinoyl glycine, captopril, hydralazine, omeprazole, gliclazide and clopidogrel, and the OTC drug *α*-lipoic acid (antioxidant). Vitamin B7 (biotin), used to improve hair and nails, interferes with insulin and C-peptide in some [9,10] immunoassays.

However, the most noteworthy of all causes is α-lipoic acid (ALA) being a drug freely available over the counter (OTC) [9]. Its nutraceutical use is to boost the concentration of the endogenously produced ALA [11,12,13,14]. The amount of ALA present in the diet is low, covalently bound and not readily available. The rationale for ALA supplementation in “health-conscious” individuals is based on its fovourable role as an exceptional antioxidant because of its unique solubility in water and lipids, hence, its efficacy as a universal free radical scavenger, a reducer of oxidative stress and as a key catalytic agent for oxidative decarboxylation of two enzymes critical to the citric acid cycle, namely, pyruvate dehydrogenase and α-ketoglutarate, through which nutrients turn into energy in mitochondria, the power house. ALA is an excellent detoxifier of metals such as copper and is also known to prolong the t_1/2_ of vitamin E (also an antioxidant) and vitamin C. ALA is available in doses up to 600 mg or as a component with other vitamins and minerals [11].

Therapeutically, ALA is prescribed for the treatment of neuropathic pain, common in diabetic patients, as well as for some neurological disorders, autoimmune diseases, vasculitis, carpal tunnel syndrome, et cetera [14,15,16]. ALA’s therapeutic potential in reducing oxidative stress and chronic inflammation continues to grow [14]. For example, ALA is promoted by some in the management of high-risk pregnancies to reduce the risk of miscarriage in the first trimester [17]. Its obstetric use is based on the desirable qualities of ALA in reducing pro-inflammatory cytokines (IL-1ß, IL-6, IL-8 and IL-17) while increasing the production of anti-inflammatory cytokine IL-10 [17] and decreasing the production of prostaglandin E2; ALA is widely advertised on UK infertility websites and recommended in online IVF forums and in books. ALA alone or in combination with others moieties such as coenzyme Q10 (ubiquinone/ubiquinol also known as a potential trigger of IAA production) [9,18,19,20] has been claim to improve fertility, reduces insulin resistance in women suffering from polycystic ovarian syndrome (PCOS), endometriosis (with N-acetyl cysteine and bromelain), infertile obese women, older women, and women with early ovarian ageing.

Hematological disorders (monoclonal gammopathy of undetermined significance (MGUS) or multiple myeloma) may produce immunoglobulins which could bind insulin, causing hypoglycaemia like IAS. However, IAS has occurred in some cases without any of the known risk factors [21,22]. Patients with other autoimmune disorders are also known to be prone to developing IAS.

The incidence of Hirata’s disease (IAS) from all causes is undoubtedly much higher in East Asians than in Caucasians, attributed to the prevalence of risk alleles in this population [2,8]. Nevertheless, it is important to point out that the first IAS case in a Caucasian was a Norwegian man and published in 1972, i.e., the same year in which Hirata published the first Japanese case in English [23]. A high number of IAS cases continued to be reported in Japanese and East Asians, with relatively few in Caucasians. For example, the first Dutch woman (Caucasian) was described in 1996 [3], while in the UK, the first case was reported as an abstract in 2010 in a patient from an East Asian background, the first British Caucasian case in a native Irish person in 2018 [24], and in a Russian in 2020 [25]. The first case of IAS in an Italian (Caucasian) appeared in Sicily (endogenous population *<* 5 million) in 2011, attributed to *α*-lipoic acid. In less than 2 years, six more cases of IAS were identified by the same medical centre in Sicily, also caused by *α*-lipoic acid, a drug available over the counter (OTC) [26]. Therefore, it may be reasonable to assume that the exact incidence of IAS in Caucasians, though low, is not certain.

The adverse impact caused by initial erroneous/misleading insulin and C-peptide measurements has been reported in hypoglycaemic cases of all ages. For example, in two cases [27,28] of children, the endocrine profiles of high serum insulin and low C-peptide legitimately raised suspicion of malicious insulin administration, prompting contact with child protection services. However, follow-up confirmatory tests established the presence of IAA, leading to the diagnosis of IAS in one (insulin/C-peptide ratio 2.8) and revealed analytical interference caused by human antimouse autoantibodies (HAMAs) in the other (insulin/C-peptide ratio 4.77). In a Chinese child (3 years old), initial findings of the insulin/C-peptide profile led to the diagnosis of hyperinsulinemic hypoglycemia; however, belated follow-up tests (OGTT) revealed the presence of IAA, thus changing the diagnosis to IAS [29]. In two other cases of secondary IAS in newborns, persistent hypoglycaemia was found to be caused by transplacental transfer of IAA from mother to fetus [7,30,31]. In a Japanese male nurse, a high insulin concentration (599 pmol/L) associated with low C-peptide (33 pmol/L) and an insulin/C-peptide ratio of 18 has raised suspicion of exogenous insulin administration. However, a test for IAA confirmed the diagnosis of IAS [32]. In two adults with hypoglycaemia, low insulin/C-peptide molar ratios (0.2 and 0.14) suggested insulinomas, which were confirmed radiologically (endoscopic ultrasound/biopsy; computed tomography); however, the subsequent findings of a high titer of IAA confirmed the co-presence of IAS in both cases [33,34].

Hyperinsulinaemia in non-diabetics is a good example because it demonstrated that initial analytical results of elevated insulin associated with low C-peptide were not accepted at face value. Further, when followed up by tests, it has confirmed erroneous measurements. Although the diagnosis was delayed and revised, it is commendable that performing follow-up tests has eventually led to the correct diagnosis and proper management.

### 3.2. Clinical Impact of an Early Error by Immunoassay in Other Cases

More important is to emphasize the universality of the inherent errors in immunoassays, meaning the underlying epitope–paratope interaction which can occur in any analysis, irrespective of the analyte’s nature. For example, initially raised oestradiol measurement by immunoassay was accepted as bona fide; this led to a patient having a hysterectomy and bilateral oophorectomy, and only when no fall in oestradiol was seen postoperatively was the sample further analysed and the original result found to be wrong [35]. Falsely high oestradiol caused mismanagement of in vitro fertilisation [36] and falsely high TSH has triggered unnecessary treatment with high doses of thyroxine for 18 months, numerous investigations and scans before the error was identified [37]. Similarly, in other cases, surgery and/or administration of chemotherapy was based on erroneous hCG measurements in blood [38,39]. Falsely elevated ACTH in a patient resulted in several invasive differential diagnostic procedures for Cushing’s disease or occult ectopic ACTH (including inferior petrosal sinus sampling), MRI and unnecessary pituitary surgery [40]. An erroneous testosterone result instigated extensive workups, including surgery for a phantom tumour producing testosterone [41]. False incongruous results were reported in troponin and B-type natriuretic peptide (BNP) in cardiac patients [42,43,44,45]. Forensically, based on a non-therapeutic very high serum digoxin level by immunoassay in a baby, a Dutch pediatric nurse was accused of administering digoxin with criminal intent, convicted and sentenced to life imprisonment; however, she was exonerated six years later when repeat analysis by an alternative non-immunoassay platform (LC-MS) revealed that the initial immunoassay result was erroneous. In the UK, three nurses were convicted of administering exogenous insulin with criminal intent and sentenced for life based on a single high insulin result associated with low C-peptide, despite an identical profile being known to occur by analytical interference and other pathologies.

In summary, the few examples of cases described above were based on the assumption that laboratory measurements by immunoassays are sufficiently reliable, hence their acceptance at face value. The incidence of fictitious results by immunoassay varied from 0.5% in the only prospective study in which the authors emphasised it is an under-estimate and up to 4% in retrospective studies [37,46,47]. The status quo that all laboratory measurements are perceived as accurate is neither tenable nor justified. The reliability of measurements prior to workup and diagnosis warrants care and could be helped and averted by appreciating the use of Bayesian logic in the interpretation of laboratory results.

Clinicians may not be aware of tests performed by immunoassays and it would be prudent that such results are indicated on the laboratory reports. Examples of tests performed by immunoassay are shown in Table 1.

## 4. Identifying Potentially Inaccurate Results by Using Bayesian Logic

Probabilistic Bayesian reasoning is a statistical concept essential for assessing confidence in immunoassay results. When interpreting these results, it is important to consider the incidence/prevalence of potential underlying pathologies. Bayes’ Theorem provides a framework for addressing this issue by allowing comparison of the relative probability of different possible explanations for an abnormal laboratory result. The following offers a simplified and intuitive overview underpinning Bayesian reasoning, focusing on the principles needed for result interpretation without relying on mathematical equations.

Bayesian reasoning will be illustrated using a contemporary example of a COVID-19 test encountered and employed during the 2020–2021 pandemic. The test used has an analytical accuracy of 98% when it was applied both to symptomatic hospitalised patients in whom the incidence/prevalence of disease was high and low in asymptomatic carriers in the community [48].

Assume that the prevalence of this condition was 80% in hospitalised patients (cohort #1) but was only 1% in the population at large (cohort #2). Applying the test to 1000 hospitalised patients would correctly identify 98% of the expected 800 patients who actually have the disease, i.e., 784 patients, and also produce 4 false-positive results (2% of the expected 200 hospitalised patients who do not have the disease); thus, the vast majority of test results in this cohort are accurate, and the probability of a false-positive test result is approximately 0.5% (4/788).

However, when the same test is applied to 1000 individuals in the community (cohort #2) where the incidence/prevalence is 1%, the same laboratory test would correctly identify the ~10 asymptomatic carriers of the disease but also produce approximately 20 false-positive results (2% of the expected 990 individuals who do not have the disease), so, in this cohort, approximately two thirds of positive laboratory results are false/inaccurate. In fact, even if an immunoassay has 100% sensitivity (ability to detect the condition under investigation when present) and 99.5% specificity (excludes this condition when absent), a significant number of positive test results (~37%) would still be false/inaccurate in this situation.

The probability that a crucial test could be false positive should be considered when the incidence/prevalence of the medical condition is low. In fact, false negatives are more common when the incidence/prevalence of the disease is high, with 2% in the above example (16 false negatives in the 800 who have the disease, cohort #1), but very small when the disease is low (2% of 10 individuals with disease in cohort #2)**.** Even if an immunoassay has 100% sensitivity (ability to detect the condition under investigation when present) and 99.5% specificity (excludes this condition when absent), a significant number of results (33.3%) would still be false/inaccurate in this situation.

## 5. Analytic; Current State

Routine laboratory biochemical and hematological tests, such as U&E, LFTs, bone profile, lipids and FBC, et cetera, can be trusted and interpreted at face value because most errors are preanalytical. Common examples are: poor phlebotomy practice, i.e., withdrawing blood after prolonged period of venous stasis with tourniquet in situ (>1 min), causing haemoconcentration, increasing all macromolecules and analytes bound to macromolecules; delays in separating serum/plasma from cells, as this form of preanalytical error occurs when blood is taken and not sent to the laboratory; and the detrimental effect of refrigerating blood for U&E tests which could increase plasma/serum potassium levels (without haemolysis) by impeding the red-cell sodium–potassium pump, repeat freeze and thawing of stored plasma/serum samples in which analytes are measured by immunoassays [49,50,51]. The above examples of preanalytical errors may not be detected or suspected by the laboratorians or clinicians, yet could be easily avoidable.

All laboratory results have reference ranges typically established by defining cut-off values that encompass 95% of measurements obtained from a healthy population. As a result, a proportion of results will fall outside the reference intervals; this inherent limitation of statistical truncation generates measurable false-positive and false-negative outcomes, the probabilities of which can be quantified through predictive values derived from the established 2 × 2 contingency table [52]. The inherently higher error rate of immunologically based tests such as immunoassays is not instigated by statistical truncation, but by the inherent nature of immunological epitope–paratope interactions (not reactions). Errors in immunoassay are intrinsic, unpredictable, variable, random and insidious.

### The Underlying Cause(s) of High Error Rate in Immunoassays

A brief background may help in appreciating the current role of immunoassays in laboratory measurements. The discovery of immunoassay as an analytical tool came about when Solomon Berson (physician) and Rosalyn Yalow (physicist), in the mid-1950s, recognised the unusual binding of labelled insulin in the serum of some patients receiving bovine/porcine insulin treatment. They also noted that labelled insulin, which bound to these endogenously produced anti-insulin antibodies in the gamma region, was displaced when unlabelled insulin was added. Such a fundamental observation heralded the birth of competitive immunoassay subsequently used to reliably measure insulin only in patients free from endogenous insulin-binding antibodies. In fact, recognising inherent fallibility of immunoassay results caused by interacting endogenous antibodies with the assay’s reagents is as old as the technique itself. The discovery of immunoassay by these two eminent scientists was awarded the Nobel Prize in 1977 to Rosalyn Yalow because Solomon Berson had died.

Immunoassay design evolved with the development of two-site excess reagent immunometric assays, i.e., labelled antibody rather than antigen. Polyclonal antibodies used as reagents are replaced by monoclonal antibodies, and non-isotopic labels such as ruthenium have replaced labelling by radioactive iodine. Analytical interference from endogenously circulating antibodies has been reported to adversely affect the accuracy of measurements in both competitive and immunometric assays, but the latter is more prone to this phenomenon. Immunoassay is considered the most significant innovation of the twentieth century, and such a successful and valuable technique that over the past four decades, the demand for tests has been exponential. This requirement has been accommodated by the development of fully automated, highly reliable, well-engineered analysers to which immunoassay methods were adapted. Immunoassay is now the largest single technology in the in vitro diagnostic (IVD) market value of 2023 at 31.3 billion USD, which was forecasted by Global Market Insights in their 2024 report to rise to 49.6 billion USD by 2032.

Immunological interactions are fallible, occurring clinically in vivo, manifesting as an autoimmune disease. In a recent study involving 22 million UK individuals, at least one autoimmune disease was found in 10% of the population [53]. Fallibility in immunoassay measurements in vitro could occur in any immunoassay test irrespective of its nature and/or format.

Reagents, standards and software for calculating results are analyser-specific, eliminating any input by the clinical laboratory in determining assay methodology. The excess-reagent immunometric assay design, coupled with reliable timing/pipetting devices, allows a non-equilibrium end point to be used. The binding reaction under these conditions follows pseudo-first-order kinetics, with antibody concentrations remaining in excess. The outcome of this formulation is a menu of assays characterised by a short assay time, excellent precision, a wide working range, increased sensitivity and exceptional repeatability. This allows tests to be carried out in a single samples, which would have been unacceptable with early manual methods, thereby containing the cost of analyses. However, such clearly favourable characteristics have overshadowed concerns about interference from circulating antibodies in some patients’ sera, which continued to be reported. In addition, some reports have minimised the seriousness of the problem or dismissed its significance altogether [54].

Errors in immunoassay caused by the presence of analytical interfering endogenous antibodies may be transient (i.e., for months following infection, immunisation, allergic reactions, blood transfusion, therapy with monoclonal antibodies, et cetera) or permanent (e.g., in autoimmune disease).

Erroneous measurement in immunoassays leading to inaccurate results is generally unique to individuals who may fortuitously have endogenous immunoglobulin antibodies and/or other moieties such as biotin/vitamin B7, capable of cross-reacting and interfering with reagents used in immunoassay analysis or signalling transduction. Each immunoassay ‘host’ presents his/her own antibody(s) as interacting ‘analytical reagents’. Because of the huge array and diversity of immunoglobulin antibodies that may be endogenously produced (around 10 billion), this form of potential interference is impossible to predict *a priori*, being confined to a specific patient’s sample, nor could it be detected by the most stringent internal or external quality control schemes [54]. Research over the last two decades has shown immunoassay error rates ranging from 0.5 to 4% [37,46,47].

## 6. Follow-Up Laboratory Tests to Detect Inaccurate/Erroneous Results

Simple and available follow-up tests are (i) serial doubling dilutions, (ii) polyethylene glycol (PEG) precipitation, (iii) repeat analyses using a different platform and (iv) the addition of native non-immune serum or heterophilic blocking antibody tubes, which is commercially available (HBT). Other tests such as liquid chromatography–mass spectrometry are excellent but not widely available in routine hospital laboratories. Aberration in any single test invalidates the measurement’s accuracy.

### 6.1. Serial Doubling Dilutions

Serial dilution of a sample to concentrations of 1:2; 1:4; 1:8 with assay diluent prior to analysis assesses the accuracy of analyses. The doubling dilutions test has the attraction of being simple; it could be performed by current automatic analysers, and it is relatively inexpensive. In this test, the behaviour of the analyte under consideration is mathematically compared with the behaviour of the results obtained in patients free from endogenous interfering antibodies [37]. This test, however, has limitations because the test could only detect inaccurate results by non-linearity/non-parallelism or even reverse linearity [37,55] in only 60% of cases. Doubling dilutions is therefore valuable only when non-linearity/non-parallelism is detected, hence, the need for an additional test if the results are not abnormal.

### 6.2. Polyethylene Glycol (PEG) Precipitation Test

PEG 6000 at 12.5% final concentration precipitates ~100% of IgM and IgG subclasses and 70% of IgA. Analysis of normal samples (controls) treated with PEG produces similar concentrations in the supernatant (≤10% reduction) while samples with interfering autoantibodies usually produce levels with >20% reductions [56,57].

### 6.3. Repeat Analysis Using Different Platform

Repeat analysis using a different immunoassay platform is commonly used. However, it is paramount to be initially aware of the biases between these two methods [56]. For example, if method A has an established positive bias of 25% relative to method B (not uncommon in some immunoassays), it would be reasonable to expect the numerical value obtained by method A to be higher than that obtained by method B. If method A gave a result 25% lower than the method B result, it would highlight that repeat analysis has revealed that something is odd, such as interference in the measurement and invalid measurements (e.g., endogenous antibodies). Even if method A gave an identical result to method B, such a repeat should still raise concern. Essentially, aberration of biases is among the telltale signs of analytical interference caused by the presence of endogenous antibodies. Failure to take method bias into account and/or failure to establish any method bias prior to clinical or forensic use could invalidate the utility and interpretation of repeat analysis using a different immunoassay platform. Repeat analysis by another method must always be objectively assessed by taking the known bias into account. It is important not to use results obtained on repeats even if they appear plausible [37].

### 6.4. Addition of Native Non-Immune Serum or Blocking Antibodies Reagent

Another commonly used alternative test is to repeat measurement before and after the addition of native non-immune serum or using the more convenient but expensive, commercially available blocking antibodies tubes (HBT) [56]. The utility of this test assumes that non-immune sera have a range of immunoglobulins, which by sheer chance may interact and neutralise endogenous interfering antibodies. A significant difference in results before and after the addition of nonimmune serum would be indicative of the presence of interference and false results. However, the utility of this test is marginal when used in conjunction with others and has a marginal effect on increasing the detection rate from interfering antibodies (3%). A better rate (20%) of increase in the detection of interference and inaccurate results can be achieved by using blocking antibodies, which increase the chance of neutralising endogenous interfering antibodies.

In summary, attention is paramount to (a) proper application of follow-up confirmatory tests and (b) their interpretation.

## 7. The Way Forward

Advances in informatics and digital transmission of data electronically from analysers to laboratory information system (LIS) and, after validation, to the patient’s electronic health record (EHR) have been introduced in most health trusts, including the UK. This creates a seamless, automated flow of diagnostic information into the patient’s digital record, thus reducing post-analysis transcription errors, enhancing and speeding timely accessibility [58,59,60,61,62,63].

Interoperability by extending algorithms in the future could highlight and integrate additional useful information such as incidence/prevalence of the disease for which the test is used and medications (prescribed and over-the-counter), which have the potential to influence laboratory analyses. These are examples which could augment the effectiveness and quality of interpretations and avert potential diagnostic misapplications by clinical practitioners. Meanwhile, in the absence of embedded patients’ electronic health care records, considerable attention is warranted when diagnosis is determined by the result of a single unverified measurement. It also makes follow-up tests for falsifications and/or additional surrogate investigations a *sine qua non* before a deterministic diagnosis is made (Table 2).

**Table 2:** Take home message.

Take Home Message
(1) Routine biochemical and hematological tests are analytically robust. Almost all errors are preanalytical and avoidable.
(2) Immunoassays are widely used for measurements of numerous analytes. Despite the relatively high analytical error rate, it is here to stay.
(3) Interpretation of immunoassay results must consider (a) accuracy of analysis (~98%) and (b) incidence/prevalence of disease for which the test is used. These considerations could identify potentially erroneous and misleading immunoassay results, avert misinterpretations, unnecessary investigations and potential diagnostic misapplications.
(4) Essentially, false-positive tests are excessive when the incidence/prevalence of disease for which the test is used is low. False-negative results are more common when the incidence/prevalence of the disease is high.
(5) Interoperability by extending algorithms could alert both clinicians and laboratorians to the need for follow-up confirmatory tests of some immunoassay measurements.

## Figures and Tables

**Table 1:** Analytes commonly measured by immunoassays.

Tests by Immunoassay Should Be Marked
All hormones
Tumour markers, e.g., carcinoembryonic antigen, prostatic-specific antigen
Specific serum proteins such as ferritin, α-fetoprotein, sex hormone binding globulin, immunoglobulins
Cardiac biomarkers such as troponin, BNP and CK-MB
Vitamin D, B12/folic acid
CRP, cytokines
Rheumatoid factor; ANA, allergy and allergen-specific IgE
Specific antibodies against bacteria/viruses, e.g., hepatitis, HIV, cytomegalovirus, rubella, syphilis, COVID-19 antibody and antigen
Anti-endocrine gland antibodies, e.g., thyroid peroxidase, thyroglobulin, intrinsic factor, adrenal
Therapeutic drug monitoring such as digoxin, gentamicin, cyclosporine
Drugs of abuse, e.g., cannabis and opiates

## Data Availability

The original contributions presented in this study are included in the article. Further inquiries can be directed to the corresponding author.
